# Endogenous immune recruitment in glioblastoma CAR T therapy: cytokine, myeloid, and chemokine circuitry

**DOI:** 10.1007/s11060-026-05497-4

**Published:** 2026-03-13

**Authors:** Justin Liu, Matthew Abikenari, Shreyas Annagiri, Joseph H. Ha, George Nageeb, Matthew Adam Sjoholm, Vaithish Velazhahan, Ravi Medikonda, John Choi, Gordon Li, Michael Lim

**Affiliations:** Department of Neurosurgery, Stanford University School of Medicine, Stanford, Palo Alto, CA 94304 USA

**Keywords:** Glioblastoma, Tumor microenvironment, Precision immunotherapy, CAR T-cell therapy, Tumor microenvironment, Endogenous immune recruitment, Myeloid reprogramming, Antigen spreading, Epitope spreading

## Abstract

**Background:**

Glioblastoma (GBM) has remained relatively unresponsive to immunotherapy, with scattered durable responses reported in early CAR T-cell studies, but without clear benefit at the population level. The major challenge for GBM has been its heterogeneous nature with a significantly immunosuppressive microenvironment that is predominantly composed of myeloid cells, inhibiting T-cell infiltration, function, and providing a rapid pathway for adaptive resistance. The focus of this review is to reposition GBM CAR T-cell therapy as a systems-level issue, turning localized CAR T-cell cytotoxicity into sustained control of the disease by engaging endogenous antitumor immunity via cytokine myeloid chemokine networks.

**Methods:**

We integrated both mechanism- and translation-oriented evidence for how inflammatory mediators derived from CAR T cells (Type I IFNs, IFN-γ, TNF) may license microglia/tumor-associated macrophages for antigen presentation and chemokine secretion, thus recruiting host effector cells and promoting antigen epitope spreading. To place this work within the context of current engineering trends, the current paper undertook a structured meta-synthesis on registry trials for interventional CAR T therapy for GBM using ClinicalTrials.gov. Using a structured advanced search strategy, we searched 91 registry records, found 44 trials for interventional CAR T therapy, and evaluated 23 active trials commenced after January 2020. Trials were classified based on target antigen choice, multi-antigen OR-gated approaches, conditional AND-gated synNotch logic, as well as safety and controllability measures (inducible off-switches).

**Conclusion:**

The effectiveness of CAR T cells for GBM is not likely to be actualized by targeting alone and needs to incorporate both killing and productive self-reinforcing endogenous immunity via myeloid licensing and chemokine amplification. Current trials are increasingly integrating this paradigm with a focus on more comprehensive antigens, gated CARs, immune-conjugate payloads, and safety designs amenable to the CNS without major toxicity such as ICANS. Future translation will require a focus on implementing endogenous immune activation as a quantified endpoint (including cytokine and chemokine analysis within CSF) and a simultaneous focus on immune set points that maintain cross-priming and memory without unmasking neuroinflammatory toxicity.

**Supplementary Information:**

The online version contains supplementary material available at 10.1007/s11060-026-05497-4.

## Introduction

Glioblastoma (GBM) is the most prevalent and aggressive primary malignant brain tumor in adults with a median overall survival rate around 12–16 months despite aggressive treatment strategies involving maximal surgical resection, radiation therapy and chemotherapy [1]. Immune checkpoint therapy that has demonstrated significant promise in melanoma and lung cancer have failed to produce meaningful clinical responses in GBM [2–4]. Given the lack of promising therapeutic approaches targeting GBM, novel treatment regimens are being explored including combining multiple checkpoint inhibitors and tumor-specific vaccines [5, 6]. However, significant barriers remain owing to the complexity of GBM necessitating the development of innovative treatment strategies.

Chimeric Antigen Receptor (CAR) T-cell therapy represents a groundbreaking approach in cancer immunotherapy leveraging advances in immune engineering and targeted tumor therapy. CAR T-cell therapy involves genetically engineering a patient’s T cells to express receptors that target specific antigens expressed on tumor cells [7, 8]. Engineering CAR T-cells begins with the extraction of the patient’s own T cells, followed by their modification using viral vectors. The resultant T cells possess an extracellular domain, usually derived from an antibody’s single-chain variable fragment (scFv), enabling them to recognize tumor antigens directly. Upon binding, these CAR T cells activate and proliferate through activation of signaling pathways associated with the intracellular CD3ζ domain and additional co-stimulatory signals from molecules such as CD28 or 4-1BB, which enhance T cell activation and persistent signaling. In essence, CAR-T cells form a non-classical immune synapse that mediate cytotoxic anti-tumor effects by engaging multiple signaling axes including perforin and granzyme, the Fas and Fas ligand pathway as well as contributing to the release of cytokines that modulate the tumor stroma [9].

CAR T-cell therapy has demonstrated significant success in hematologic malignancies, particularly targeting CD19 in B-cell neoplasms. Approved therapies include tisagenlecleucel (Kymriah) for B-cell acute lymphoblastic leukemia and axicabtagene ciloleucel (Yescarta) for large B-cell lymphoma [9, 10]. In recent years, there has been emerging interest in applying CAR T-cell therapy to various solid tumors, including GBM, with ongoing clinical trials exploring various targets beyond CD19 [8, 11, 12].

While CAR T-cell therapies have shown promise in treating hematological cancers, several challenges persist in their application for effective treatment of GBM. A major hurdle is antigen escape, where tumor cells may downregulate or lose the target antigen after therapy initiation, leading to resistance [13–17]. Tumor heterogeneity in GBM leads to varying, context-dependent antigen expression within a single tumor, complicating effective targeting [18–20]. Furthermore, the immunosuppressive microenvironment of GBM, enriched with myeloid-derived suppressor cells and regulatory T cells, further inhibits T cell activity [21–24]. T cell exhaustion, characterized by diminished cytotoxic functionality following prolonged exposure to antigen, presents a significant barrier [25, 26].

To achieve durable clinical responses, it is essential to engage not only CAR T cells but also the host’s endogenous immune system. Combining CAR T-cell therapy with strategies that recruit and activate other immune cells, for instance by enrichment of CAR T-cells with immune stimulatory cytokines, could augment the overall antitumor response [27, 28]. This synergistic interaction can promote long-term remission and facilitate antigen spreading [29, 30], supporting the maintenance of immune pressure on tumor cells and preventing relapse.

Our review will address the challenges of CAR T-cell monotherapy in treating GBM and elucidate strategies for enhancing CAR T-cell efficacy through recruitment and activation of endogenous immune cells. Augmenting the recruitment of endogenous immune cells may provide a pathway for enhancing the design and application of CAR T-cell therapies in the complex landscape of glioblastoma to improve efficacy and sustained remission in patients.

In addition to summarizing the mechanistic and translational literature, we conducted a structured meta-synthesis of CAR T-cell clinical trials in glioblastoma using publicly available registries to provide context for the current development pipeline. Utilizing a prespecified keyword approach to search the ClinicalTrials.gov database, we screened 91 articles and identified 44 interventional trials of CAR T in patients with GBM, ultimately selecting 23 trials initiated after January 2020 for our analysis. This analysis was conducted in order to provide context for trial motifs of contemporary design, such as those involving novel target antigens, multi-antigen or OR-gated strategies, SynNotch/AND-gated approaches, and inducible safety strategies.

## CAR-T therapy in hematologic vs. solid tumors

### Hematologic malignancies

CAR-T cell therapy has delivered unprecedented remission rates in B-cell malignancies like acute lymphoblastic leukemia (ALL) and non-Hodgkin lymphoma [31–33]. CAR-T cells targeting CD19 have induced deep, long-term remissions, with many patients remaining disease-free years after treatment [34–36]. Similarly, anti-BCMA CAR-T cells for multiple myeloma have achieved durable responses, including multi-year progression-free survival in a subset of patients [37]. These outcomes suggest that CAR-T cells can provide sustained immune surveillance against blood cancers. Notably, the host immune system appears to contribute to these lasting remissions [37]. CAR-T cells secrete inflammatory cytokines (e.g. IFN-γ) and cause tumor lysis, which can recruit and activate endogenous immune effectors. The recruitment of the host immune system secondary to CAR-T cell-mediated killing is termed antigen/epitope spreading [38, 39]. In leukemia and lymphoma, which typically lack a profound immunosuppressive niche, such host immunity engagement may eliminate antigen-negative escape variants and reinforce the therapeutic effect. Longitudinal studies in myeloma patients found that sustained remissions were associated with robust CAR-T expansion *and* a preserved repertoire of host T cells, alongside low levels of suppressive myeloid cells [37, 40]. This interplay between engineered T cells and the patient’s immune system underscores how CAR-T therapy in hematologic malignancies can orchestrate a broad, lasting anti-tumor immune response.

### Solid tumors

In solid cancers, CAR-T therapy has faced major hurdles due to the tumor’s physical and immunosuppressive barriers. Unlike disseminated blood malignancies, solid tumors form dense masses that are hard for T cells to penetrate [41–44]. Fibrotic stroma, abnormal vasculature, and extracellular matrix components create physical hurdles that impede CAR-T cell infiltration [45–47]. Moreover, the tumor microenvironment (TME) actively suppresses anti-tumoral immune activity through a variety of suppressive mechanisms. Tumor and stromal cells often upregulate immune checkpoint ligands such as PD-L1, which engage PD-1 on T cells to blunt their activity [48–50]. They also exploit metabolic immune evasion: indoleamine 2,3-dioxygenase 1 (IDO1) is frequently expressed, depleting tryptophan and accumulating kynurenine – conditions that starve and paralyze effector T cells [51–53]. Additionally, malignant and stromal cells secrete immunosuppressive cytokines like TGF-β, and chemokines that recruit regulatory T cells (Tregs) and myeloid-derived suppressor cells (MDSCs) into the tumor [54–56]. These components of the TME collectively form a milieu that suppresses T cell–mediated cytotoxicity. The net effect is that CAR-T cells in solid tumors often become functionally exhausted or excluded before eliminating the cancer. Consistent with this, clinical studies report that CAR-T cells exhibit poor expansion and persistence in solid tumor patients compared to hematologic settings [15, 57]. Thus, the immunologic landscape of solid tumors, marked by physical exclusion and active immune suppression (via PD-L1, IDO1, TGF-β pathways and others), remains a fundamental barrier to replicating the success of CAR-T seen in blood cancers.

Lastly, an important factor for why CAR therapy has shown the most reproducible efficacy so far in hematologic malignancies is that the target expression is more uniform and clonally preserved compared with most solid malignancies. Antigens like CD19, CD20, CD22, and BCMA are more universally and highly expressed on malignant cells, thereby minimizing the effects of intratumoral antigen heterogeneity and the likelihood of developing antigen loss variants early on. Also, malignant cells are more easily accessible compared with solid malignancies, thereby overcoming significant barriers that make CAR therapy ineffective for solid malignancies [15].

## Lessons from CAR-T therapy

An emerging principle in CAR-T therapy is the importance of antigen spreading for durable cancer control. The resulting inflammatory tumor cell death from CAR-T cell therapy can expose a breadth of tumor antigens to the immune system. This can activate new anti-tumor T cell responses beyond the CAR’s target specificity [39, 58]. Additionally, CAR-T cells in these patients often persist as memory-like cells, providing ongoing surveillance. In solid tumors, however, the development of secondary immune responses is curtailed by the suppressive TME. The presentation of released antigens to T cells can be inefficient due to dysfunctional antigen-presenting cells and inhibitory signals in the TME. The contrast is clear: in liquid cancers, CAR-T therapy can catalyze a wider endogenous immune attack and enduring remission, whereas in solid tumors the lack of immune propagation means that CAR-T cells bear the full therapeutic burden. Future CAR-T strategies are therefore focusing on increasing inflammation in the TME (i.e. “immune hot”) by combining CAR-T cells with checkpoint inhibitors or cytokine modulation to promote infiltration, inflammation, and epitope spreading within solid tumors [59–62]. By learning from the immune dynamics of successful CAR-T cases, researchers aim to engineer therapies that not only directly kill tumor cells but also reshape the host-tumor interaction, bridging the efficacy gap between hematologic and solid cancers.

## Mechanisms of endogenous immune recruitment

Despite CAR T-cells targeting specific antigens, recruitment of the endogenous immune system is essential for achieving a robust therapeutic response in GBM. Cytokines are key immunomodulatory molecules that play a central role in this process, enabling and enhancing CAR T-cell antitumor activity by mobilizing and activating endogenous immune populations. Interferon-γ (IFN-γ) is a key pro-inflammatory and anti-tumoral cytokine that shapes endogenous myeloid responses by promoting macrophage polarization toward an M1 phenotype and enhancing dendritic-cell antigen presentation through upregulating MHC classes I and II [63]. IFN-γ signaling is also critical for cross-talk between CAR T-cells and the host immune system. This bidirectional cytokine exchange is central to therapeutic efficacy.

In addition, interferon-γ also directly regulates endogenous immune cell recruitment through a chemokine and adhesion gene program to facilitate the trafficking of immune cells to the tumor. In a tumor microenvironment, IFN-γ signaling can increase the expression of ligands of CXCR3 chemokines, such as CXCL9, CXCL10, and CXCL11, as well as adhesion molecules such as ICAM-1/VCAM-1, to facilitate effector T cell and NK cell infiltration [64, 65]. In a GBM microenvironment, in which overcoming blood-brain barriers as well as immune cell exclusion represents a major barrier, this inflamed response induced by IFN-γ secretion can potentially increase this microenvironment’s permeability to a certain degree, shifting it from a poorly permeable to a relatively permeable microenvironment to facilitate a higher degree of antigen presentation following cell death induced by CAR T cell cytolysis [63, 65, 66]. Nevertheless, chronic IFN-γ signals can induce adaptive resistance gene expression, such as PD-L1 expression, to potentially suppress efficacy as well as provide a rationale to combine IFN-γ with strategies to overcome checkpoint blockade resistance.

In syngeneic GBM models, IFN-γ was required not only for CAR T cell–mediated tumor control but also for activating endogenous immune populations to mount complementary antitumor responses [65]. Interleukin-12 (IL-12) and interleukin-18 (IL-18) are proinflammatory cytokines that synergize to amplify innate antitumor immunity. Together, they activate and recruit NK cells, enhance NK cytotoxicity, and help overcome Treg-mediated suppression. Activated NK cells then produce high levels of IFN-γ, which drives macrophage polarization toward the proinflammatory M1 phenotype and further suppresses Tregs [67, 68]. T cells redirected for antigen-unrestricted cytokine-initiated killing (TRUCKs) are CAR T-cells that can release cytokines to modulated the tumor microenvironment (TME) [69]. TRUCKs enable CAR T cells to remodel the tumor microenvironment and generate productive inflammation, thereby recruiting endogenous immune cells and promoting antitumor activity even against tumor cells not directly targeted by the CAR.

TRUCKs have been engineered to secrete IL-12, thereby amplifying IFN-γ production, enhancing the antitumor activity of T cells and NK cells, and suppressing Treg function [70]. In GBM models utilizing EGFRvIII specific CAR T-cells, IL-12 coadministration augmented treatment efficacy. This combination also activated endogenous immune populations, shifting the TME from immunosuppressive to proinflammatory by reducing CD4⁺ Tregs and increasing CD4⁺ effector T cells. IL-12 further expanded intratumoral myeloid cells, which the authors proposed contributed to a more inflammatory TME through enhanced antigen presentation [71]. TRUCKs engineered to release IL-18 have shown success in treating pancreatic and lung tumors that were previously unresponsive to CAR T therapy without supportive cytokines. IL-18 secretion was associated with increased inflammatory macrophage and NK-cell populations, along with reduced Tregs and immunosuppressive dendritic cells. In contrast, IL-12 and IL-21 were less effective at eliciting these responses in the models tested [70, 72]. Although these findings are promising, IL-18–based TRUCKs have not yet been thoroughly investigated in the context of GBM.

Interleukin-2 (IL-2) is essential for the proliferation and survival of activated T cells and NK cells. High levels of IL-2 support the expansion of effector lymphocytes and can even reverse Treg-mediated suppression of immune responses [67, 73]. IL-2 has been explored as an adjunct in CAR T-cell therapy. In patients receiving EGFRvIII-targeted CAR T cells (NCT01454596), coadministration of intravenous IL-2 produced limited clinical benefit [74]. Similarly, IL13Rα2-targeted CAR T cells engineered to resist glucocorticoids and delivered with intracranial IL-2 induced only modest responses in patients with GBM [75], suggesting that IL-2 may provide some support but is insufficient as a standalone adjuvant. Overall, the therapeutic value of IL-2 coadministration in CAR T-cell treatment for GBM remains unclear.

Tumor necrosis factor-α (TNF-α) is a potent pro-inflammatory cytokine that promotes tumor cell apoptosis and drives inflammatory responses by binding to its cell-surface receptors and activating programmed cell-death pathways [76]. In glioma-bearing mice, treatment with IL13Rα2-targeted CAR T-cells increased intratumoral IFN-γ and TNF-α levels and expanded CD8⁺ T cells, which in turn promoted dendritic-cell trafficking into the CNS and helped establish an inflammatory TME. Notably, this CAR T monotherapy did not reduce Treg populations [77]. Similarly, in a human patient with recurrent GBM (NCT02208362), IL13Rα2 CAR T-cell therapy induced increases in IFN-γ, TNF-α, and IL-2 but produced only a transient clinical response [16].

Granulocyte macrophage-colony stimulating factor (GM-CSF) is known for supporting myeloid pro-inflammatory activity and is secreted by a broad range of cells [78, 79]. In murine ovarian tumor models, CAR T cells engineered to secrete GM-CSF and IFN-γ increased the abundance of tumor-associated macrophages, which contributed to enhanced antitumor activity [80]. To our knowledge, this effect has not yet been demonstrated in GBM.

In addition to cytokine-mediated support of endogenous immunity, CAR T cells can promote antigen spread, thereby facilitating a broader antitumor response beyond the specific target antigen. Antigen spread occurs when tumor cells are lysed, releasing a diverse array of tumor-associated antigens that can be captured and presented by antigen-presenting cells, ultimately activating additional endogenous immune populations. This mechanism may help explain how CAR T cells directed against a single antigen can induce a more generalized antitumor response [81–83]. Together, cytokine-driven immune recruitment and antigen spread highlight the capacity of CAR T-cell therapies to reshape the broader antitumor immune landscape.

From the perspective of cytokine support in CAR T-cell therapy, tertiary lymphoid structures (TLSs) represent a promising mechanism for amplifying cytokine-mediated antitumor responses. TLSs are organized aggregates of lymphoid and stromal cells that arise in chronically inflamed tissues, and their presence within tumors is generally associated with improved prognosis [84, 85]. TLSs have been identified in human gliomas, and because the brain parenchyma lacks lymph nodes to support localized immune activation, these structures may serve as focal sites for antigen presentation and immune-cell priming [85]. Sabahi et al. describe an approach to generate inducible TLSs using an implantable biodegradable scaffold designed to retain antigen-containing tissue and recruit immune cells, thereby promoting TLS formation. Once established, such TLSs could provide sustained, localized immune support to enhance CAR T-cell activity [86]. This strategy offers a novel means of counteracting the immunosuppressive GBM TME by fostering sustained, site-specific antitumor immunity. Such an approach could further augment CAR T-cell therapies that rely on cytokine release to generate a broader and more durable antitumor response. Table 1 recapitulates the endogenous immune recruitment program initiated during GBM CAR T therapy.

**Table 1:** Endogenous immune recruitment program initiated during GBM CAR T therapy: measurable mediators and cellular shifts

Program element	What increases / decreases	Mechanistic interpretation in GBM CAR T	Evidence-based emphasis	Practical readouts
Tumor inflammation after CAR T activity	Increased inflammatory mediators, including IFN-γ and TNF-α	CAR T activity promotes an inflammatory microenvironment that can support endogenous immune engagement	Preclinical and clinical observations (IL13Rα2 CAR T; patient and mouse data)[87]	CSF inflammatory mediators after locoregional CAR T; intratumoral cytokine increases
Myeloid activation and antigen presentation capacity	Increased activated macrophage/microglia phenotype (CD86 + MHC-IIhigh)	IFN-γ from CAR T cells promotes myeloid activation that supports endogenous T-cell function	Immune-competent murine GBM model described; supportive logic across Sect. [88]	Tumor myeloid activation markers (CD86, MHC-II) in post-treatment tissue
Chemokine program linked to Th1 inflammation	Increased CXCL9 and CXCL10 (and associated Th1-polarized signals)	Chemokines are consistent with the recruitment/positioning of endogenous immune cells, even if trafficking is not always demonstrated in tissue	Locoregional IL13Rα2 trial CSF analysis; discussion of Th1 polarization [87]	CSF CXCL9/CXCL10 elevations after infusion
Endogenous CD8 + T-cell activation	Increased intratumoral endogenous CD8 + T cells with increased IFN-γ, granzyme B, Ki-67	CAR T therapy can be associated with activation/expansion of endogenous effector T cells	EGFRvIII CAR T trial findings; mechanistic support from murine model [15]	Tumor markers of activation/proliferation (IFN-γ, granzyme B, Ki-67)
Antigen/epitope spreading	Increased breadth of tumor antigens presented after lysis (conceptual increase in antigen availability)	Tumor cell lysis increases antigen availability, enabling broader endogenous responses beyond the CAR target	Described mechanistically, framed as important for durable control [30]	Tumor/immune activation patterns consistent with broader immunity (narrative endpoint)
Adaptive resistance / counter-regulation	Increased FOXP3 + Tregs and increased PD-L1, TGF-β, IL-10; increased exhausted T cells (PD-1high/TIM-3+)	Endogenous activation often co-occurs with compensatory suppression that limits durability	EGFRvIII CAR T trial; EGFRvIII + PD-1 blockade trial at relapse [89]	Post-treatment tumor profiling: Tregs, checkpoint ligands, exhaustion markers

## Immune cell population regulation during CAR T therapy

Clinical trials in GBM evaluating CAR T cells indicate that these therapies not only directly attack tumor cells but also modulate the patient’s endogenous immune compartment by upregulating and downregulating various lymphocyte populations. For example, O’Rourke and colleagues conducted a 10-patient phase I study of EGFRvIII-CAR T cells (NCT02209376), which showed expansion of endogenous CD8^+^ T cells within the tumor. This was accompanied by markers of activation and proliferation, namely IFN-γ, granzyme B, and Ki-67. Concurrently, there was a substantial increase in FOXP3^+^ Tregs and elevated expression of immunosuppressive molecules such as TGF-β, PD-L1, and IL-10 within the tumor along with persistence of myeloid infiltration, suggesting an adaptive resistance response to CAR T therapy [15].

In a separate phase I trial evaluating locoregional delivery of IL13Rα2 CAR T cells (NCT01082926), cerebrospinal fluid showed transient elevations in IFN-γ and associated chemokines CXCL9, CXCL10, and IL-12, consistent with a Th1-polarized inflammatory response following CAR T infusion [75]. Although CXCR3^+^ immune cells, such as CD8^+^ and NK cells, are known to be responsive to these chemokines, the study did not demonstrate increased bystander immune cell trafficking into tumor tissue.

Another recent phase I trial of EGFRvIII-targeted CAR T cells in newly diagnosed GBM combined with CAR T therapy with PD-1 blockade, which did not result in objective clinical responses [90]. In paired tumor analyses before treatment and at relapse, investigators observed a post-therapy enrichment of immunosuppressive T cell subsets, specifically an increased infiltration of FOXP3^+^ Tregs and a higher prevalence of exhausted T cells. These changes, identified through in situ phenotyping and single-cell transcriptomic profiling of resected tumor tissue, demonstrated that post-CAR T specimens contained more PD-1^high^/TIM-3^+^ T cells indicative of T cell exhaustion and expanded Treg populations compared to pre-treatment samples.

Preclinical studies provide mechanistic support for these clinical findings by showing that CAR T-secreted IFN-γ remodels the GBM microenvironment and activates endogenous antitumor immunity. In an immune-competent murine model, IFN-γ produced by CAR T cells drove activation of intratumoral macrophages and microglia (CD86^+^MHC-II^high^), which in turn supported expansion and functional licensing of endogenous CD8^+^ T cells characterized by Ki-67 and granzyme expression [88]. Importantly, endogenous T cells isolated after CAR treatment displayed tumor-specific cytotoxicity, and durable rejection of antigen-negative rechallenge tumors required both CAR-derived IFN-γ and host IFNγR signaling, establishing causality between CAR T cytotoxic activity, IFN-γ-mediated myeloid activation, and the endogenous CD8^+^ expansion observed in clinical trials. Table 2 summarizes strategies that aim to improve GBM CAR T durability by enhancing endogenous immunity or limiting adaptive resistance.

**Table 2:** Strategies that aim to improve GBM CAR T durability by enhancing endogenous immunity or limiting adaptive resistance

Strategy class	What increases / decreases	Rationale in GBM	Representative examples
Cytokine support / TRUCKs	Increased local inflammatory cytokines (e.g., IL-12 or IL-18); increased endogenous innate/effector activation; decreased Treg influence in supportive models	Cytokines can remodel the TME toward proinflammatory programs and support recruitment/activation of endogenous cells	TRUCKs; IL-12 augmentation in EGFRvIII models with decreased CD4 + Tregs and increased CD4 + effectors; IL-18 TRUCK activity in non-GBM solid tumor models (not yet fully tested in GBM)
Locoregional delivery	Increased CNS-compartment bioactivity (increased CSF inflammatory mediators such as IFN-γ, CXCL9, CXCL10)	GBM enables compartment-resolved cytokine programs; locoregional routes can generate measurable CNS immune activation	IL13Rα2 locoregional phase I experience with CSF inflammatory mediator increases; intratumoral/intraventricular delivery as design principle
Multi-antigen targeting	Decreased antigen-escape vulnerability; increased coverage of heterogeneous antigen expression	Antigen heterogeneity and immunoediting are central failure e modes in GBM	Bivalent EGFR/IL13Rα2 strategies; multivalent concepts discussed in Engineering/Future Directions
Engager-secreting platforms	Increased recruitment/engagement of non-engineered T cells; decreased dependence on a single antigen	Reach beyond the CAR logic to address heterogeneity and recruit bystanders	CARv3 TEAM E; BiTE-secreting CAR logic grounded in preclinical GBM work and first-in-human report
Prime-and-kill circuits	Increased specificity and increased ability to address heterogeneous antigen expression	Programmable recognition to reduce reliance on static single-antigen targeting	SynNotch-induced CAR programs; first-in-human trial mention (NCT06186401)
Combination strategies to preserve host immunity	Increased probability of sustaining antigen spreading and endogenous responses; decreased adaptive resistance (conceptual goal)	Checkpoint blockade framed as preserving/amplifying host immunity rather than rescuing CAR T alone; radiation and STING to increase immunogenicity	Checkpoint blockade discussion; radiation synergy concept; STING agonism remodeling in murine GBM/human explants
Toxicity management as enabling design	Decreased CRS/ICANS severity when managed promptly; decreased neuroinflammatory complications	CNS inflammatory programs must be harnessed without unacceptable toxicity	Tocilizumab for CRS in hematologic settings; steroids for ICANS; steroids and IL1R blockade noted in CSF-delivered bivalent CAR T neurotoxicity context; intracranial CRS concept

**Fig. 1:**
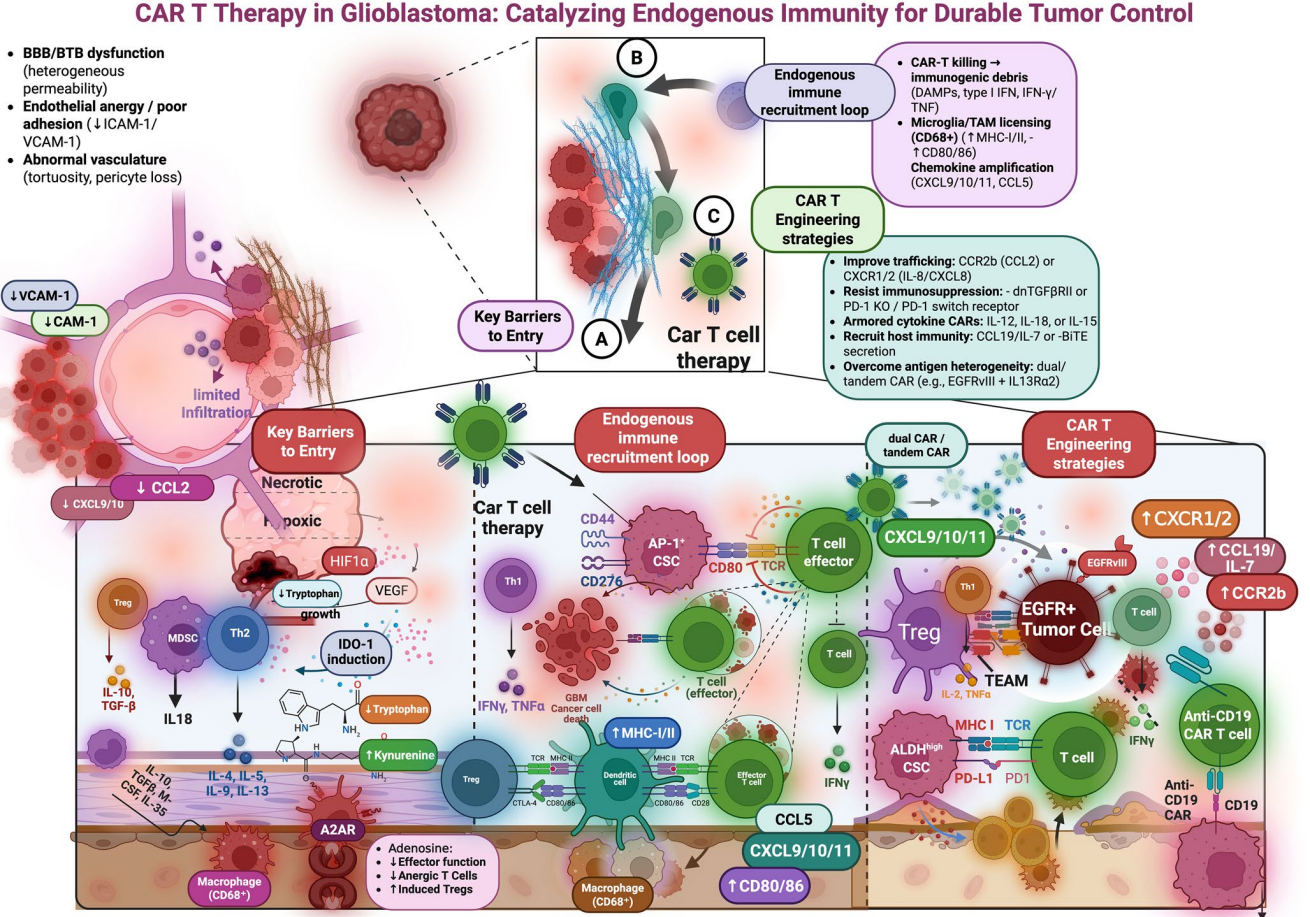
CAR T therapy in glioblastoma can be reframed as a two-part problem: overcoming entry barriers and amplifying endogenous immunity through engineered reinforcement loops. (**A**) Key barriers to CAR T entry and function in the GBM microenvironment. Variable BBB/ BTB permeability, endothelial cell anergy with downregulated adhesion molecules, abnormally structured vasculature, and high extracellular matrix density restrict T cell entry. Following entry, the hypoxia/necrosis pathways (HIF-1α to VEGF), adenosine pathways, and populations of immunosuppressive myeloid and regulatory lymphocytes, such as Tregs and suppressive myeloid cells, limit effector cell function by secreting cytokines (IL-10, TGF-β), inducing metabolic stress, and expressing inhibitory ligands. (**B**) Endogenous immune recruitment loop initiated by CAR T cytotoxicity. CAR T-induced tumor cell death results in immunogenic debris (DAMPs and inflammatory cytokines such as type I interferons, IFN-γ, and TNF), which can license microglia/TAMs for antigen presentation (upregulate MHC-I/II and co-stimulation via CD80/86). This licensing enables chemokine amplification (CXCL9/10/11 and CCL5), which recruits and retains host effector cells (CD8 + T cells and NK cells), thereby enhancing overall antitumor immunity (epitope spreading) beyond the original CAR target. (**C**) Engineering strategies to convert CAR T activity into durable, system-level tumor control. Some examples of modular designs are: trafficking (CCR2b, CXCR1/2); immunosuppressive escape (dominant-negative TGF-β receptor signaling or PD-1 reversing receptors); armored cytokine circuits (IL-12, IL-18, IL-15); recruitment of the endogenous immune response (CCL19/IL-7 or T-cell engagers); and multi-target designs to overcome the issue of antigen heterogeneity (two-target tandem CAR designs). These approaches are designed to convert the immune landscape in GBM from one that is immune-excluded and myeloid-dominant state toward a chemokine-rich, antigen-presenting milieu that supports coordinated tumor clearance. Figure made with Biorender (Dec 2025). Abbreviations: A2AR, adenosine A2A receptor; ALDH, aldehyde dehydrogenase; AP-1, activator protein-1; BBB, blood–brain barrier; BiTE, bispecific T-cell engager; BTB, blood–tumor barrier; CAR, chimeric antigen receptor; CCL, C-C motif chemokine ligand; CCR2b, C-C chemokine receptor 2b; CSC, cancer stem cell; CXCL, C-X-C motif chemokine ligand; CXCR, C-X-C chemokine receptor; DAMPs, damage-associated molecular patterns; dnTGFβRII, dominant-negative transforming growth factor-β receptor II; EGFR, epidermal growth factor receptor; EGFRvIII, EGFR variant III; HIF-1α, hypoxia-inducible factor-1α; ICAM-1, intercellular adhesion molecule-1; IDO-1, indoleamine 2,3-dioxygenase-1; IFN, interferon; IL, interleukin; IL13Rα2, interleukin-13 receptor alpha 2; MDSC, myeloid-derived suppressor cell; MHC, major histocompatibility complex; NK, natural killer; PD-1, programmed cell death protein 1; PD-L1, programmed death-ligand 1; TCR, T-cell receptor; TEAM, T-cell–engaging antibody molecule; TAM, tumor-associated macrophage; TGF-β, transforming growth factor-β; Th1/Th2, T helper 1/2; TNF, tumor necrosis factor; Treg, regulatory T cell; VCAM-1, vascular cell adhesion molecule-1; VEGF, vascular endothelial growth factor

In summary, CAR T therapy in GBM patients can upregulate endogenous effector immune elements, such as cytotoxic T cells and activated myeloid cells, along with proinflammatory mediators, but this immune activation frequently coincides with the induction of counter-regulatory mechanisms, such as Treg accumulation and checkpoint ligand expression, that may limit efficacy. These insights, derived from early-phase trials with small patient numbers, are largely correlative and must be interpreted cautiously. Further clinical studies are needed to confirm these immune alterations and to determine how to tip the balance toward a more robust, durable anti-GBM immune response in the context of CAR T therapy. Figure 1 recapitulates the Key barriers to CAR T entry and function in the GBM microenvironment, as well as the emerging mechanisms of endogenous immune in CAR-T therapy.

## Clinical trial landscape (registry meta-synthesis)

### Methods: meta-synthesis from CAR-T clinical trials in GBM

**Fig. 2:**
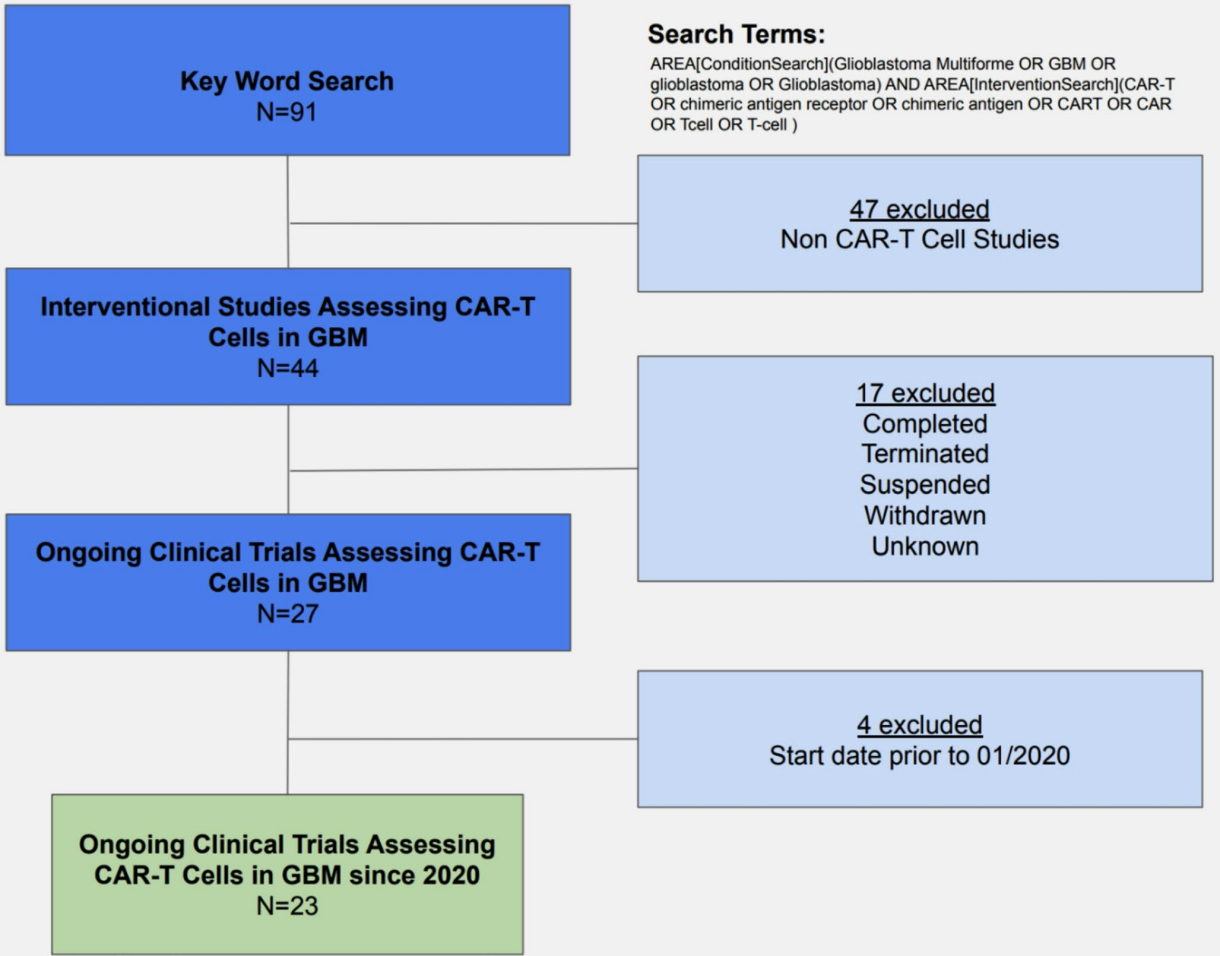
ClinicalTrials.gov trial selection workflow for CAR T-cell studies in glioblastoma

We undertook a structured landscape review (meta-synthesis) of interventional clinical trials of chimeric antigen receptor (CAR) T-cell therapy in glioblastoma (GBM) using the ClinicalTrials.gov trial registry. The search strategy utilized the advanced search features of the ClinicalTrials.gov trial registry and the search query: AREA[ConditionSearch](Glioblastoma Multiforme OR GBM OR glioblastoma OR Glioblastoma) AND AREA[InterventionSearch](CAR-T OR chimeric antigen receptor OR chimeric antigen OR CART OR CAR OR Tcell OR T-cell). Figure 2 recapitulates our search strategy.

Study identification and screening: The keyword search yielded 91 registry entries. Excluding 47 trials not involving CAR T-cell therapies, 44 interventional trials involving CAR T-cell therapies for GBM were obtained. The trials were further filtered to include ongoing trials, leaving 27 trials. Excluding trials not ongoing, according to registry status (Completed, Terminated, Suspended, Withdrawn, or Unknown), 17 trials were removed. The final exclusion involved removing 4 ongoing trials that started before 01/2020, leaving 23 ongoing trials for CAR T-cell therapies for GBM started after 2020.

Eligibility Criteria: Trials had to fulfill all of the following criteria to be included: (1) interventional study design, (2) GBM included in the condition/diagnosis criteria, (3) CAR T-cell therapy included in the intervention criteria, (4) registry status indicating that the trial is currently ongoing at the time of the screening (e.g. Recruiting, Active not recruiting, Not yet recruiting, Enrolling by invitation), and (5) start date on or after 01/2020. Criteria for the exclusion of trials included non-CAR T-cell interventions, non-interventional study designs for the main landscape, non-ongoing studies, and start dates prior to 01/2020.

Data Extraction: For each trial included, we abstracted the registry-reported variables into a standardized table that included the NCT number, trial phase, start date, primary completion date, trial title, trial status, condition(s), age criteria, and intervention description.

Synthesis Method: As we sought to describe rather than combine efficacy results, we performed a descriptive meta-synthesis. Trials were characterized by (i) target antigen(s), (ii) multi-antigen strategies (bispecific or dual-targeting strategies), (iii) conditional logic-gating strategies (AND-gating by synNotch), and (iv) safety and controllability components (inducible suicide or off-switches), as recorded in the registry intervention and title entries, Table 3.

**Table 3:** Summary of ongoing clinical trials in glioblastoma

NCT Number	Phases	Start Date	Primary Completion Date	Study Title	Study Status	Conditions	Age	Interventions
NCT06815432	PHASE1	7/3/25	6/5/29	GPC-3 CAR T CELLS FOR Recurrent GPC-3 Positive Glioblastoma	NOT_YET_RECRUITING	Glioblastoma	Adult	15.GPC3-CAR T cells
NCT06616727	PHASE1	12/26/23	12/31/24	The Safety and Efficacy of SNC-109 CAR-T Cells Therapy the rGBM	ENROLLING_BY_INVITATION	Recurrent Glioblastoma Multiforme (GBM)	Adult	SNC109
NCT05366179	PHASE1	9/2/22	5/30/30	Autologous CAR-T Cells Targeting B7-H3 in Recurrent or Refractory GBM CAR.B7-H3Tc	RECRUITING	Glioblastoma	Adult	CAR.B7-H3T cells infusion
NCT07193628	PHASE1|PHASE2	9/26/25	12/31/27	B7H3/IL13Ra2 Bispecific Armored Chimeric Antigen Receptor T-Cell Therapy Study for Recurrent/Refractory Glioblastoma	NOT_YET_RECRUITING	Refractory Glioblastoma|Recurrent Glioblastoma	Adult	EPC-003 Fully Human Anti-B7H3/IL13Ra2 Armored CAR-T Cell Therapy
NCT05474378	PHASE1	7/12/22	2026-08	B7-H3 Chimeric Antigen Receptor T Cells (B7-H3CART) in Recurrent Glioblastoma Multiforme	Recruiting	Glioblastoma	Adult	B7-H3CART
NCT05353530	PHASE1	7/25/23	2027-12	IL-8 Receptor-modified CD70 CAR T Cell Therapy in CD70 + Adult Glioblastoma	Recruiting	Glioblastoma	Adult	Ex-Vivo expanded autologous IL-8 receptor (CXCR2) modified CD70 CAR (8R-70CAR) T cells
NCT05627323	PHASE1	6/6/23	2025-10	CAR T Cells in Patients With MMP2 + Recurrent or Progressive Glioblastoma	ACTIVE_NOT_RECRUITING	Glioblastoma	Adult	CHM-1101 CAR-T cells
NCT05802693	PHASE1	11/15/23	11/14/25	A Study to Evaluate the Safety, Tolerance and Initial Efficacy of EGFRvIII CAR-T on Glioblastoma	NOT_YET_RECRUITING	Recurrent Glioblastoma	Adult	Targeted Epidermal Growth Factor Receptor Variant III(EGFRvIII) autochimeric antigen receptor T cell injection
NCT06691308	PHASE1	11/12/24	5/31/27	WL276 CAR-T Cell Therapy for CD276 Positive Recurrent or Progressive Glioblastoma Patients	NOT_YET_RECRUITING	Recurrent or Progressive Glioblastoma	Adult	WL276 CAR-T cells
NCT04214392	PHASE1	2/26/20	8/31/25	Chimeric Antigen Receptor (CAR) T Cells With a Chlorotoxin Tumor-Targeting Domain for the Treatment of MMP2 + Recurrent or Progressive Glioblastoma	ACTIVE_NOT_RECRUITING	Recurrent Glioblastoma|Recurrent Malignant Glioma|Recurrent WHO Grade II Glioma|Recurrent WHO Grade III Glioma	Adult	Chlorotoxin (EQ)-CD28-CD3zeta-CD19t-expressing CAR T-lymphocytes (via ICT delivery)
NCT05577091	PHASE1	9/30/23	11/1/24	Tris-CAR-T Cell Therapy for Recurrent Glioblastoma	Recruiting	Recurrent Glioblastoma	Adult	Inverse correlated dual-target, truncated IL7Ra modified CAR -expressing autologous T-lymphocytes.
NCT05241392	PHASE1	1/27/22	11/30/24	Safety and Efficacy Study of Anti-B7-H3 CAR-T Cell Therapy for Recurrent Glioblastoma	ACTIVE_NOT_RECRUITING	Glioblastoma	CHILD, ADULT	B7-H3-targeting CAR-T cells
NCT05835687	PHASE1	4/27/23	2028-03	Loc3CAR: Locoregional Delivery of B7-H3-CAR T Cells for Pediatric Patients With Primary CNS Tumors	Recruiting	Central Nervous System Neoplasms|Atypical Teratoid/Rhabdoid Tumor|Diffuse Midline Glioma, H3 K27M-Mutant|Ependymoma|High Grade Glioma|Glioblastoma|Medulloblastoma	Adult	B7-H3-CAR T cells
NCT04661384	PHASE1	3/5/21	11/17/25	Brain Tumor-Specific Immune Cells (IL13Ralpha2-CAR T Cells) for the Treatment of Leptomeningeal Glioblastoma, Ependymoma, or Medulloblastoma	ACTIVE_NOT_RECRUITING	Ependymoma|Glioblastoma|Medulloblastoma|Recurrent Metastatic Malignant Neoplasm in the Leptomeninges	Adult	IL13Ralpha2-specific Hinge-optimized 41BB-co-stimulatory CAR Truncated CD19-expressing Autologous T-Lymphocytes
NCT05168423	PHASE1	2/24/23	12/19/39	CART-EGFR-IL13Ra2 in EGFR Amplified Recurrent GBM	ACTIVE_NOT_RECRUITING	Glioblastoma	Adult	CART-EGFR-IL13Ra2 Cells
NCT06186401	PHASE1	4/30/24	8/31/26	Anti-EGFRvIII synNotch Receptor Induced Anti-EphA2/IL-13Ralpha2 CAR (E-SYNC) T Cells	Recruiting	EGFR Gene Mutation|Glioblastoma|MGMT-Unmethylated Glioblastoma|Recurrent Glioblastoma	Adult	E-SYNC T Cells
NCT05660369	PHASE1	3/22/23	1/1/26	CARv3-TEAM-E T Cells in Glioblastoma	Recruiting	Glioblastoma|Malignant Glioma|Recurrent Glioblastoma|Recurrent Glioma	Adult	CARv3-TEAM-E T cells
NCT06973096	PHASE1	7/18/25	2042-07	CART-EGFR-IL13Ra2 in Newly Diagnosed GBM Following Initial Radiotherapy	RECRUITING	Glioblastoma	Adult	CART-EGFR-IL13Ra2 cells
NCT06815029	PHASE1	6/17/25	10/11/30	Intracranial Genetically Modified Immune Cells (TGFŒ≤R2KO/IL13RŒ±2 CAR T-Cells) for the Treatment of Recurrent or Progressive Glioblastoma or Grade 3 or 4 IDH-Mutant Astrocytoma	Recruiting	Recurrent Astrocytoma, IDH-Mutant, Grade 3|Recurrent Astrocytoma, IDH-Mutant, Grade 4|Recurrent Glioblastoma	Adult	Chimeric Antigen Receptor T-Cell Therapy
NCT07180927	PHASE1|PHASE2	9/10/25	12/31/28	DLL3 CAR-T Therapy Targeting Brain Tumors	Recruiting	Glioblastoma of Cerebellum	CHILD, ADULT, OLDER_ADULT	4SCAR DLL3 T cells
NCT06764537	Observational	2025-04-03	2029-04-05	Evaluation of in Vitro Antitumor Activity of GD2 CAR-T Cells in Glioblastoma	Not Yet Recruiting	Glioblastoma	Adult	Blood collection
NCT06946680	PHASE1	3/18/25	2030-12	IL-8 Receptor-modified CD70 CAR T Cell Therapy in CD70 + Pediatric High-grade Glioma (HGG)	Recruiting	High-grade Glioma	CHILD, ADULT	Ex-Vivo expanded autologous IL-8 receptor (CXCR2) modified CD70 CAR (8R-70CAR) T cells
NCT06018363	PHASE1|PHASE2	6/1/23	12/31/27	Clinical Study on the Treatment of Malignant Brain Glioma by QH104 Cell Injection	Recruiting	Brain Gliomas|High-Grade Gliomas|GBM	Adult	Allogenic B7-H3 CAR-Œ≥Œ¥T cell(QH104)

## Results

Chimeric Antigen Receptor (CAR) T cell therapy presents as a unique challenge in glioblastoma therapy, with the low mutational burden of the tumor often cited as the most contributive limitation due the reduced generation of neoantigens that may serve as potential targets for a CAR. Additionally, the substantial cellular and antigen heterogeneity present within an individual GBM tumor as well as within a population of GBM patients has hampered the ability to engineer a consistently efficacious CAR. Indeed, while some notable CAR-T cell trials for GBM in the past decade have demonstrated long term responses in 1–2 patients, statistically meaningful results for larger cohort sizes have been virtually nonexistent.

Following a decade of CAR-T cell clinical trials often targeting a handful of antigens with modest results, next generation CAR-T cells employ a range of novel techniques aimed at boosting efficacy and addressing the well established limitations of previous generation CAR T cells. In the 23 identified ongoing CAR -T cell trials in GBM, these approaches primarily include targeting of newly established antigens with greater percent expression in GBM cells, dual targeting mechanisms/OR gating for increased CAR range, synNotch receptors/AND gating for the targeting and localization of CARs to the TME, and rapidly inducible safety switches to minimize toxicities.

The extensive search for well confined antigens expressed in a substantial portion of GBM cells but not in normal brain parenchyma has paved the way for a host of targets for next generation CAR-T cells, of which B7-H3 (CD276) is the most studied. B7-H3 is an immune checkpoint molecule that, unlike PD-1, CTLA-4, or LAG3, is enriched in tumor cells (particularly glioma stem cells) but not cells of the normal brain. This distinction enables B7-H3 as a promising target for a CAR, as investigated in clinical trials such as NCT06691308, NCT05241392, NCT05835687, NCT05366179, NCT07193628, NCT05474378, and NCT06691308, of which the majority are phase 1, dose escalation studies with cohort sizes under 50. An additional target of interest for next generation CARs is matrix metalloproteinase (MMP2). MMP2 is an enzyme with expression strongly associated with glioma stem cell markers and highly expressed at infiltrative tumor margins. MMP2 naturally binds in complex with chlorotoxin (CLTX), allowing for CLTX expressing CAR T cells to have strong specificity for MMP2. Trials such as NCT04214392 and NCT05627323 investigate this CAR-T cell mechanism in recurrent or progressive GBM, with cohort sizes of *N* = 19 and *N* = 52 respectively.

Beyond the identification of novel targets, next generation CARS employ dual targeting mechanisms (‘OR’ gating) to increase the range of antigens a CAR may recognize and kill, potentially improving the efficacy of CARs particularly in the context of GBM, where established antigens are often variably expressed in the tumor environment or localized to specific cellular subtypes. NCT07193628 is an ongoing dose escalation trial investigating the potential of a bispecific CAR for B7-H3 and IL13Ra2 antigens in recurrent or refractory GBM, with a current cohort size of 14 patients. Of note, in this trial, the dual targeting approach involved combining a less established, ‘next generation CAR’ B7-H3 antigen with a historically trialed ‘previous generation CAR’ antigen IL13Ra2. Other trials such as NCT05168423 and NCT06973096 employ a bi-specific CAR for previously established EGFRvIII and IL13Ra2 antigens, likely with the goal of determining if targeting both antigens may rescue the modest therapeutic benefits demonstrated from previous generation therapies that targeted these antigens individually. One trial, NCT05577091, is investigating a bispecific CAR targeting glioma stem cell antigens CD44 and CD133 in recurrent GBM, with a current cohort size of 10.

In addition to bi-specific CARs which enable a broader range of antigen recognition, the development of ‘AND’ gating via syn-Notch receptors is perhaps the most advanced frontier in CAR-T cell therapy and has enabled CAR-T cells to have dramatic levels of specificity to the tumor microenvironment. Trial NCT06186401 is currently investigating the safety and ideal dose for a EGFRVIII synNotch receptor induced EphA2/IL-13Ra2 bispecific CAR T cell. Such a design ensures that the expression of the bispecific EphA2/IL-13Ra2 CAR occurs only following a binding and recognition event between the CAR synthetic notch receptor and the EGFRVIII antigen in the tumor microenvironment, allowing not only for the benefits in CAR range from the bispecific receptor, but also an increased degree of tumor localization prior to activation, potentially improving efficacy and minimizing systemic effects of CAR administration.

Concurrent to improvements in CAR targeting and binding to antigens, several advances in next generation CARs have focused on reducing toxicity following administration of therapy, such as through inducible ‘off switches’ for administered CAR T cells. Most notable is the icasp9 suicide switch, as employed in NCT06815432, which enables rapid killing of the administered CAR T cells if therapy results in severe toxicity or additional side effects.

### CRS, ICANS and TIAN

Cytokine release syndrome (CRS) and immune effector cell-associated neurotoxicity syndrome (ICANS) are two major toxicities observed in CAR T-cell therapy clinical trials. Mechanistically, CAR T cells become activated upon encountering their target antigen and secrete inflammatory cytokines such as IFNγ and TNFα, which in turn stimulate host monocytes and macrophages to release IL-1, IL-6 and other mediators [91]. Clinically, CRS manifests as a systemic hyperinflammatory response characterized by symptoms ranging from fever and fatigue to circulatory shock, vascular leakage, disseminated intravascular coagulation, and multi-organ system failure [92]. In CD19 CAR T trials for B-cell malignancies (NCT01626495, NCT01029366, NCT01044069, NCT01593696, NCT02028455), the majority of patients experienced CRS, with a substantial subset developing high-grade (≥ 3) CRS [34, 35, 93, 94]. An IL-6 receptor–blocking antibody, tocilizumab, has become a standard intervention that often rapidly reverses CRS symptoms within hours without compromising CAR T efficacy [16, 95]. By contrast, in CAR T studies for GBM, severe systemic CRS has been infrequent. Only isolated “intracranial CRS” events have been noted. For example, a case report of IL13Rα2-targeted CAR T intracranial infusion noted mild fever and cytokine elevations limited to the CNS [16].

One small GBM CAR T trial targeting EphA2 did observe two patients with Grade 2 CRS (fever and pulmonary edema) (NCT03423992), but these symptoms resolved with dexamethasone and no other organ toxicities occurred [96]. In contrast to these typically mild events, a recent phase I trial of GD2-directed CAR T cells in children with diffuse midline gliomas, including DIPG, reported CRS in 15 of 20 patients, with six experiencing grade ≥ 3 toxicity marked by fever, hypotension, and systemic cytokine elevations following intravenous infusion [97, 98]. This study stands out as a rare example of high-grade CRS in a solid tumor setting, likely reflecting the immunologically active perivascular environment of midline brain tumors and the robust expansion of CAR T cells following systemic delivery.

Although often overlapping with CRS, Immune Effector Cell-Associated Neurotoxicity Syndrome (ICANS) represents a distinct neurotoxic syndrome characterized by CNS dysfunction rather than systemic inflammation. Unlike CRS which primarily affects peripheral organs and vasculature, ICANS manifests with neurologic symptoms driven by cytokine-mediated disruption of the blood-brain barrier and endothelial activation [99]. In the aforementioned GD2-CAR T trial, neurotoxicity was particularly evident in patients receiving intraventricular infusions, where symptoms included but were not limited to somnolence, encephalopathy, and increased intracranial pressure, alongside imaging evidence of localized peritumoral edema [97]. Inflammatory toxicities such as CRS and ICANS are markedly rarer in GBM CAR T cell trials compared to hematologic malignancies. While most studies, such as those targeting EGFRvIII and IL13Rα2, report only mild, localized symptoms like transient fever or headache, isolated cases of moderate CRS have been observed, such as in an EphA2-directed trial, confirming that these events, though uncommon, can still occur in glioma patients [16, 96]. In hematologic malignancies, ICANS is far more prevalent. In the pivotal ZUMA-1 trial evaluating axi-cel (NCT02348216), a CD19-directed CAR T cell therapy for the treatment of refractory large B-cell lymphoma, ICANS occurred in 67% of patients, with 32% experiencing grade ≥ 3 events such as encephalopathy, aphasia, and somnolence, typically emerging around five days post-infusion [100].

ICANS management in trials relies on intensive supportive care and corticosteroids to diminish inflammation, with dexamethasone often favored for its high CNS penetration [101]. These clinical findings support that while CRS and ICANS are dominant toxicities in hematologic CAR T therapies, their occurrence in GBM is significantly less frequent and often limited to low-grade, localized manifestations. Nonetheless, the potential for neuroinflammatory complications, particularly with intracranial delivery, necessitates stringent neuromonitoring in glioma patients receiving CAR T therapy and should inform the design of future clinical trials aimed at optimizing both safety and efficacy for solid CNS tumors.

Lastly, in addition to CRS and ICANS, there has also been the emergence of a distinct and local toxicity phenomenon termed tumor inflammation-associated neurotoxicity (TIAN), which has been noted with the use of cell therapies targeted at CNS tumors [102]. Unlike CRS and ICANS, which are defined by the presence of systemic inflammation and more generalized encephalopathy, respectively, TIAN refers to the tumor-related and regionally specific phenomenon of neuroinflammation that can manifest with transient episodes of neurologic deterioration and/or inflammation-related edema, hydrocephalus, and increased intracranial pressure, depending on the tumor location and extent. Notably, TIAN subsumes the phenomenon of pseudoprogression but is more inclusive than edema, particularly because inflammatory signals can also mediate neurologic manifestations even when there is limited or no significant tumor progression evident on neuroimaging. A TIAN grading system has also been proposed to provide standardized reporting and guidance on the management and monitoring of patients with CNS tumor immunotherapy clinical trials, particularly with the use of intracranial administration or with high-risk neuroanatomic sites that are more susceptible to inflammatory mass effects.

## Therapeutic implications

Glioblastoma forces CAR T-cell therapy to function not only as a cytotoxic drug but as an in situ immune-reprogramming platform, because the tumor microenvironment is typically myeloid-dominant and immunosuppressive, with tumor-associated macrophages/microglia shaping T-cell dysfunction and poor antigen presentation [103].

### Combination strategies

A central translational lesson from early GBM CAR T experience is that on-target activity can be coupled to rapid immunologic counter-adaptation. In an EGFRvIII-directed CAR T trial, CAR T cells trafficked to tumor sites and EGFRvIII expression decreased in post-treatment specimens, yet the post-infusion tumor milieu showed increased expression of inhibitory molecules and greater regulatory T-cell infiltration, consistent with adaptive immune resistance that could blunt both CAR T and endogenous T-cell contributions [15]. Therefore, checkpoint blockade is a compelling strategy in GBM when framed as a strategy to preserve or amplify antigen spreading–driven host T-cell immunity rather than as a “rescue” for CAR T cells alone. Clinical checkpoint data in GBM remain mixed: neoadjuvant anti–PD-1 therapy produced measurable pharmacodynamic immune remodeling and was initially associated with improved survival in recurrent GBM, while subsequent work highlights heterogeneity and resistance pathways that likely constrain generalized benefit, supporting biomarker-guided, mechanism-matched combinations with CAR T [104, 105].

Radiation is also foundational in GBM management, and its immunologic value is increasingly viewed through the lens of antigen release, vascular/TME remodeling, and cross-priming potential. Recent preclinical work using intravital approaches supports true synergy between radiation and CAR T activity in an immunocompetent glioblastoma setting, strengthening the argument that priming the tumor niche can make CAR T cells better recruiters of endogenous immunity rather than solitary effectors [106]. Concurrently, STING pathway activation is attractive in GBM because it can engage innate sensing programs and reshape the local cytokine landscape. In murine GBM models and human explants, a STING agonist triggered inflammatory outputs and remodeled the tumor microenvironment with prominent innate immune engagement, motivating rational CAR T pairings aimed at converting GBM into a more immunogenic, cross-presenting state [107].

A key practical implication of locoregional GBM CAR T trials is that CNS-compartment cytokine programs are measurable and therapeutically meaningful. In the largest completed IL-13Rα2 CAR T phase I experience (65 recurrent high-grade glioma patients; most recurrent GBM) (NCT02208362), locoregional dosing was feasible and associated with CNS increases in inflammatory mediators including IFNγ and CXCL9/CXCL10, aligning with a chemokine axis that could recruit and position endogenous effector cells [87]. The challenge is to harness these programs without unacceptable neuroinflammation. “Armoring” approaches that conditionally deliver supportive cytokines or remodel suppressive myeloid populations are particularly relevant in GBM, where MDSCs and TAMs constrain durable T-cell function. For example, IL-15 modification has been reported to enable CAR T cells to function as a dual-targeting agent against tumor cells and MDSCs in GBM models, conceptually aligning cytokine support with endogenous immune recruitment rather than simple CAR T persistence [108].

### Engineering approaches

GBM clinical signals increasingly suggest that the most durable advances will come from engineering CAR T cells to broaden recognition and actively recruit non-engineered immune effectors, while building in safeguards for CNS toxicity. Locoregional administration is no longer a niche tactic in GBM but rather is becoming a design principle. IL13Rα2 CAR T cells delivered intratumorally/intraventricularly achieved acceptable safety with clear CNS cytokine bioactivity and a subset of meaningful disease control [87]. The phase I intrathecal bivalent EGFR/IL13Rα2 CAR T trial (NCT05168423) enrolled 18 patients with recurrent GBM and represents a deliberate attempt to broaden antigen recognition while leveraging CSF delivery. This strategy produced frequent radiographic tumor regression but was accompanied by substantial rates of grade 3 neurotoxicity, underscoring that effective trafficking and tumor engagement must be paired with careful control of CNS inflammation [108].

Antigen heterogeneity and immunoediting remain central failure modes in GBM CAR T therapy, motivating multi-antigen and reach beyond the CAR designs [15]. While single-antigen CARs can induce meaningful tumor regression, selective pressure frequently results in downregulation or loss of the targeted epitope, leaving antigen-negative clones to drive recurrence. One strategy to address this limitation involves engineering CAR T cells to secrete bispecific T-cell engagers (BiTEs). BiTEs are antibody-based molecules that simultaneously bind CD3 on endogenous T cells and a tumor-associated antigen, thereby redirecting non-engineered T cells toward tumor cells independent of CAR expression. By recruiting bystander T cells against additional tumor antigens, BiTE-secreting CAR platforms extend tumor coverage beyond the original CAR target and reduce dependence on uniform antigen expression [109].

One approach designed to address antigen heterogeneity while preserving tumor specificity is the use of synthetic Notch (SynNotch) receptor systems. SynNotch receptors are engineered, modular receptors that trigger a programmable transcriptional response only after recognition of a defined “priming” antigen. In CAR T applications, this architecture enables a prime-and-kill circuit where initial engagement of a tumor-restricted antigen activates expression of a secondary receptor targeting tumor-associated antigens. This conditional logic spatially restricts CAR activity to the tumor microenvironment while expanding antigen coverage beyond a single static epitope and has demonstrated efficacy in GBM-relevant contexts [110]. The emergence of first-in-human SynNotch-inspired GBM trials (e.g., EGFRvIII SynNotch–induced multi-antigen CAR programs) highlights how recruitment of endogenous immunity can be coupled to *programmable recognition* rather than relying on a single static antigen (NCT06186401).

While EGFRvIII was initially considered an attractive target due to its tumor-specificity, accumulated clinical trial data reveal fundamental limitations. EGFRvIII expression is subclonal and spatially heterogeneous, with antigen loss consistently documented following CAR T exposure [14, 89]. These findings establish that EGFRvIII-directed monotherapy is insufficient to durably alter tumor progression. The next generation of GBM CAR targets must address this heterogeneity through three integrated approaches. First, multi-antigen targeting strategies, such as bispecific CARs, synNotch logic-gated circuits, and T-cell engaging platforms (BiTEs and TEAMs), can mitigate antigen escape by broadening recognition beyond single markers [109]. Second, prioritizing antigens with higher tumor penetrance and more uniform expression, such as B7-H3 which is expressed in > 75% of GBMs with enrichment in glioma stem cells [111], may provide more durable coverage. Third, integrating single-cell transcriptomics and spatial proteomics can identify surface antigens conserved across malignant cellular states [112] and resistant to immunotherapeutic selection pressure, representing promising paths toward targets that maintain expression under immune pressure (Table 4).

**Table 4:** Major antigens targeted in contemporary GBM CAR T Trials

Antigen	Biological Rationale	Number of Ongoing Trials
B7-H3 (CD276)	Type I transmembrane immune checkpoint protein that promotes immune evasion. Significantly overexpressed in gliomas [111]	7 trials: NCT05366179, NCT05474378, NCT06691308, NCT05241392, NCT06018363, NCT07193628 (bispecific with IL13Rα2), NCT05835687 (includes GBM in pediatric CNS trial)
IL13Rα2	Decoy receptor that sequesters IL-13; highly overexpressed in GBM (particularly mesenchymal subtype) with minimal normal tissue expression; associated with aggressive phenotype [106, 113]	6 trials: NCT04661384, NCT06815029, NCT05168423 (bispecific with EGFR), NCT06973096 (bispecific with EGFR), NCT06186401 (synNotch-induced with EphA2), NCT07193628 (bispecific with B7-H3)
EGFRvIII	Tumor-specific deletion mutation of EGFR creating constitutively active receptor; present in around 30% of newly diagnosed GBM; completely tumor-specific with zero normal tissue expression [14, 110]	3 trials: NCT05802693, NCT06186401 (synNotch priming receptor), NCT05660369 (CARv3-TEAM-E)
CD70	TNF superfamily costimulatory molecule; aberrantly expressed in gliomas; normally restricted to activated lymphocytes; promotes tumor survival and immune suppression when overexpressed [114]	2 trials: NCT05353530 (adult GBM), NCT06946680 (pediatric HGG)
CD44/CD133	Cancer stem cell markers; CD44 is hyaluronan receptor involved in invasion and stem cell maintenance; CD133 enriched in glioma stem cell populations resistant to standard therapy [20]	1 trial: NCT05577091 (bispecific targeting both)

## Future directions

GBM is now entering a phase where progress will be determined less by whether CAR T cells can reach the brain, and more by whether they can (i) quantify and induce host immunity, (ii) overcome spatial/antigenic heterogeneity, and (iii) establish durable immune memory without intolerable neuroinflammation. A near-term priority is to operationalize “endogenous immune recruitment” as a measurable clinical endpoint, not an inferred mechanism. GBM uniquely enables longitudinal sampling via CSF reservoirs, creating a practical route to track: (1) TCR repertoire dynamics (CSF and blood), (2) cytokine/chemokine programs that predict recruitment (for example IFNγ–CXCL9/10 patterns seen after locoregional IL-13Rα2 CAR T), and (3) tumor antigen burden and immunoediting. Clinical trials are already pointing to implementable correlates. Intrathecal bivalent EGFR/IL13Rα2 CAR T treatment produced substantial CAR T abundance and cytokine release in CSF in all treated patients in an interim analysis, alongside early-onset neurotoxicity managed with steroids and IL1R blockade, illustrating both the opportunity and the need for compartment-resolved immune monitoring. Finally, “liquid biopsy” approaches in CSF are becoming mechanistically informative: CARv3-TEAM-E reports included CSF-based molecular tracking aligned with rapid radiographic changes, suggesting a path to integrate antigen escape surveillance with endogenous immune readouts in real time.

GBM heterogeneity is not simply an antigen-selection problem; it is an immune-engagement problem.

When CAR T pressure eliminates an antigen-positive compartment, the remaining tumor may be both antigen-divergent and more suppressive. This is clinically foreshadowed by EGFRvIII CAR T data showing both antigen decrease and a shift toward an inhibitory, Treg-enriched environment after infusion. Future work should treat heterogeneity as a design constraint that requires two coupled solutions: (i) broadened recognition (bivalent/multivalent CARs, prime-and-kill circuits) and (ii) deliberate recruitment of non-engineered effectors (e.g., engager-secreting platforms). The intracerebroventricular bivalent EGFR(epitope 806)/IL-13Rα2 CAR T phase I trial (18 treated patients) provides a recent clinical anchor for this approach, showing tumor regression in a majority of patients with measurable disease, but also high rates of grade 3 neurotoxicity, emphasizing that broader targeting must be paired with control of CNS inflammation [108].

Durable GBM control likely requires a transition from an acute CAR T response to a self-renewing endogenous immune state, potentially involving sustained antigen presentation, myeloid repolarization, and development of functional memory T-cell pools. Mechanistically, IFNγ has emerged as a key mediator linking CAR T activity to host myeloid activation and induction of endogenous immunity, providing a concrete axis to optimize rather than treating immune recruitment as a black box [88]. However, the same inflammatory circuitry that may enable memory can also drive neurotoxicity. The field therefore needs “immune rheostats”: tunable cytokine programs, on-switches/off-switches, and rational anti-inflammatory co-therapies that preserve cross-priming while preventing runaway CNS inflammation, an issue underscored by the neurotoxicity profiles in CSF-delivered bivalent CAR T trials.

Lastly, in parallel, AI-enabled phenotyping is likely to become a practical lever for GBM CAR T development by improving patient stratification and trial learning. For one, routine pathology and imaging-based prediction models will enable immune states and predictions for response to immunotherapy to inform trials [115]. These predictions will enable pre-treatment estimates for heterogeneity for antigens, myeloid cell dominance, and adaptive resistance programs to inform CAR-T trials. In the perioperative period, tumor segmentation and surgical planning will enable a more precise relationship between tumor and imaging phenotypes, immune cell states in CSF, and toxicity, including TIAN [15, 116–119].

## Electronic Supplementary Material

Below is the link to the electronic supplementary material.

Supplementary Material 1

## Data Availability

No datasets were generated or analysed during the current study.
