# Supplementary material for: Endogenous immune recruitment in glioblastoma CAR T therapy: cytokine, myeloid, and chemokine circuitry
Source: J Neurooncol. 2026 Mar 13;177(1):50. doi: 10.1007/s11060-026-05497-4 (PMC12982237; doi:10.1007/s11060-026-05497-4)
Supplement: Supplementary file 1 — Supplementary Material 1 [file 11060_2026_5497_MOESM1_ESM.docx]

**Supplemental table 1. Study sponsors and treatment mode of administration**

| **NCT Number** | **Study Title** | **Sponsor** | **Mode of administration** |
| --- | --- | --- | --- |
| NCT06616727 | The Safety and Efficacy of SNC-109 CAR-T Cells Therapy the rGBM | Shanghai Simnova Biotechnology Co.,Ltd. | Not explicitly stated |
| NCT06018363 | Clinical Study on the Treatment of Malignant Brain Glioma by QH104 Cell Injection | Dushu Lake Hospital Affiliated to Soochow University | Ommaya reservoir or intrathecal administration |
| NCT05366179 | Autologous CAR-T Cells Targeting B7-H3 in Recurrent or Refractory GBM CAR.B7-H3Tc | UNC Lineberger Comprehensive Cancer Center | Intraventricular infusion |
| NCT05168423 | CART-EGFR-IL13Ra2 in EGFR Amplified Recurrent GBM | University of Pennsylvania | Intrathecal administration |
| NCT06973096 | CART-EGFR-IL13Ra2 in Newly Diagnosed GBM Following Initial Radiotherapy | University of Pennsylvania | Intracerebroventricular injection |
| NCT05353530 | IL-8 Receptor-modified CD70 CAR T Cell Therapy in CD70 + Adult Glioblastoma | University of Florida | Intravenous infusion |
| NCT05660369 | CARv3-TEAM-E T Cells in Glioblastoma | Marcela V. Maus, M.D.,Ph.D. | Ommaya reservoir |
| NCT05474378 | B7-H3 Chimeric Antigen Receptor T Cells (B7-H3CART) in Recurrent Glioblastoma Multiforme | Stanford University | Intracerebroventricular (ICV) administration or both ICV and intratumoral administration |
| NCT06946680 | IL-8 Receptor-modified CD70 CAR T Cell Therapy in CD70 + Pediatric High-grade Glioma (HGG) | University of Florida | Intravenous infusion |
| NCT05627323 | CAR T Cells in Patients With MMP2 + Recurrent or Progressive Glioblastoma | Chimeric Therapeutics | Intracerebroventricular and intratumoral administration |
| NCT06815432 | GPC-3 CAR T CELLS FOR Recurrent GPC-3 Positive Glioblastoma | Baylor College of Medicine | Intracavitary administration |
| NCT06764537 | Evaluation of in Vitro Antitumor Activity of GD2 CAR-T Cells in Glioblastoma | Central Hospital, Nancy, France | N/A |
| NCT07193628 | B7H3/IL13Ra2 Bispecific Armored Chimeric Antigen Receptor T-Cell Therapy Study for Recurrent/Refractory Glioblastoma | Second Affiliated Hospital, School of Medicine, Zhejiang University | Intraventricular injection |
| NCT05241392 | Safety and Efficacy Study of Anti-B7-H3 CAR-T Cell Therapy for Recurrent Glioblastoma | Beijing Tiantan Hospital | Intracavitary or Ommaya reservoir |
| NCT06186401 | Anti-EGFRvIII synNotch Receptor Induced Anti-EphA2/IL-13Ralpha2 CAR (E-SYNC) T Cells | Hideho Okada, MD, PhD | Intravenous infusion |
| NCT05577091 | Tris-CAR-T Cell Therapy for Recurrent Glioblastoma | Beijing Tiantan Hospital | Intratumoral or intraventricular administration using Ommaya reservoir |
| NCT05802693 | A Study to Evaluate the Safety, Tolerance and Initial Efficacy of EGFRvIII CAR-T on Glioblastoma | Beijing Tsinghua Chang Gung Hospital | Omaya capsule |
| NCT07180927 | DLL3 CAR-T Therapy Targeting Brain Tumors | Shenzhen Geno-Immune Medical Institute | Intravenous infusion |
| NCT06691308 | WL276 CAR-T Cell Therapy for CD276 Positive Recurrent or Progressive Glioblastoma Patients | Beijing Immunochina Medical Science & Technology Co., Ltd. | Intracranial administration using Ommaya reservoir |
| NCT04661384 | Brain Tumor-Specific Immune Cells (IL13Ralpha2-CAR T Cells) for the Treatment of Leptomeningeal Glioblastoma, Ependymoma, or Medulloblastoma | City of Hope Medical Center | Intracerebroventricular administration |
| NCT06815029 | Intracranial Genetically Modified Immune Cells (TGFŒ≤R2KO/IL13RŒ±2 CAR T-Cells) for the Treatment of Recurrent or Progressive Glioblastoma or Grade 3 or 4 IDH-Mutant Astrocytoma | City of Hope Medical Center | Intracranial administration |
| NCT04214392 | Chimeric Antigen Receptor (CAR) T Cells With a Chlorotoxin Tumor-Targeting Domain for the Treatment of MMP2 + Recurrent or Progressive Glioblastoma | City of Hope Medical Center | Intracerebroventricular and intratumoral administration |
| NCT05835687 | Loc3CAR: Locoregional Delivery of B7-H3-CAR T Cells for Pediatric Patients With Primary CNS Tumors | St. Jude Children's Research Hospital | CNS reservoir catheter |
